# The Value of a Panel of Autoantibodies for Predicting the Activity of Lupus Nephritis at Time of Renal Biopsy

**DOI:** 10.1155/2015/106904

**Published:** 2015-02-26

**Authors:** Gabriella Moroni, Silvana Quaglini, Antonella Radice, Barbara Trezzi, Francesca Raffiotta, Piergiorgio Messa, Renato Alberto Sinico

**Affiliations:** ^1^Division of Nephrology, Fondazione Ospedale Maggiore, Mangiagalli, Regina Elena, 20122 Milano, Italy; ^2^Department of Informatics, Università degli Studi di Pavia, 27100 Pavia, Italy; ^3^Institute of Microbiology, Azienda Ospedaliera, Ospedale San Carlo Borromeo, 20153 Milano, Italy; ^4^Renal Unit and Clinical Immunology Unit, Azienda Ospedaliera, Ospedale San Carlo Borromeo, 20153 Milano, Italy

## Abstract

Few studies have correlated serum biomarkers with renal histology, the gold standard for renal activity, in lupus nephritis (LN). We tested a panel of autoantibodies and complement at the time of kidney biopsy and after treatment. Anti-dsDNA, anti-nucleosome, anti-ribosome P, and anti-C1q antibodies and C3/C4 were measured in 107 patients with LN at the time of renal biopsy and after 6–12 months and were correlated with clinical/histological parameters. At multivariate analysis, high titers of anti-C1q antibodies or of anti-dsDNA antibodies (*P* = 0.005, OR = 8.67, CI: 2.03–37.3) were the independent predictors that discriminate proliferative from nonproliferative LN. All the immunological parameters, except anti-ribosome, showed a significant correlation with activity index but not with chronicity index. Only anti-C1q showed a significant correlation with the amount of proteinuria (*R* = 0.2, *P* = 0.03). None of the immunological parameters were predictive of remission at 6 and 12 months. We found that anti-C1q alone or in combination with anti-dsDNA emerged as the most reliable test in differentiating proliferative and nonproliferative LN. Anti-C1q was the only test correlated with the clinical presentation of LN. After treatment, the titre of the autoantibodies was significantly reduced, but none was predictive of remission.

## 1. Introduction

Lupus nephritis (LN) is one of the most frequent manifestations of Systemic Lupus Erythematosus (SLE) and represents a major determinant of disease morbidity and mortality [1]. Its clinical course is often characterized by flares of activity alternated with periods of quiescence, generally induced by therapy [2]. The identification of noninvasive biomarkers may help to predict the renal involvement at diagnosis and monitor relapses of LN during the follow-up. Many studies have tested the value of a number of autoantibodies for predicting or confirming the diagnosis of renal flares with contrasting results. Some [3–5] but not all studies [6] have demonstrated that anti-dsDNA antibodies (anti-dsDNA) and complement fractions may be useful in assessing the disease and the renal activity. One paper [7] and a recent review [8] concluded that anti-nucleosome antibodies have high prevalence in severe LN but are of limited help in differentiating active from inactive LN. A number of cross sectional studies found that antiC1q antibodies (antiC1q) have a significant association with renal involvement [9–15]. In our previous paper on a large cohort of SLE patients evaluated prospectively for 6 years, we demonstrated that renal exacerbations seem to be quite improbable in the presence of normal values of C3, C4, anti-dsDNA, anti-C1q, and that anti-C1q was slightly better than the other tests to confirm the clinical activity of LN [16].

Noteworthy, in the vast majority of studies the diagnosis of LN flares relies on variable clinical definitions based on activity of urine sediment, amount of proteinuria, and deterioration of renal function, whilst the “gold standard” for the diagnosis of renal activity is represented by renal biopsy. In this prospective study, serum samples at renal biopsy and after the induction therapy of 107 LN patients were tested for a panel of autoantibodies (including anti-dsDNA, anti-C1q, anti-nucleosome, anti-ribosome antibodies, and C3 and C4 complement fractions) to investigate their association with the clinical and histological data.

## 2. Patients and Methods

One hundred and seven patients with SLE, diagnosed according to the American College of Rheumatology criteria [17] (94 females, 13 males) at admission in two Italian Renal Units (Fondazione Ospedale Maggiore and Azienda Ospedaliera Ospedale San Carlo Borromeo, Milano) to undergo renal biopsy for assessment of LN, entered the study. The renal biopsies were classified following the ISN/RNP classification [18]. Activity and chronicity indices were calculated according to Austin et al. [19].

Sera at renal biopsy were tested for a panel of auto antibodies including anti-dsDNA and anti-C1q, anti-nucleosome, and anti-ribosome antibodies as well as C3 and C4 complement fractions.

The study does not need an ethical approval. We have obtained an informed consent to participate in the study from all the patients involved.

### 2.1. Objectives

The aim of this study was to assess the performance of these tests in predicting:

- the histological classes of lupus nephritis,
- the activity and chronicity index at renal biopsy,
- the clinical feature of LN at renal biopsy,
- the response of lupus nephritis at 3, 6, and 12 months after the beginning of the induction therapy.

### 2.2. Laboratory Investigations

Anti-dsDNA antibodies were measured by a commercial quantitative ELISA (Varelisa anti-dsDNA Antibodies, Phadia GmbH, Freiburg, Germany) and C3 and C4 plasma levels by nephelometry (Nephelometer Analyser II, Behring, Marburg GmbH, Germany).

Anti-C1q antibodies were detected using a* home-made* ELISA as described by Sinico et al. [11].

Anti-nucleosome antibodies were measured by ELISA according to manufacturer instructions using Quanta Lite Chromatin assay (INOVA diagnostics, Inc., San Diego, CA, USA). [20].

Anti-ribosome P antibodies were measured by ELISA according to manufacturer instructions using Quanta Lite Ribosomal P assay (INOVA diagnostics, Inc., San Diego, CA, USA).

### 2.3. Definition of Activity

At each clinical examination the activity of LN was classified as follows [2]:

 0 = complete renal remission: normal renal function for at least 6 months, proteinuria <0.5 g/24 h, and urinary red blood cells <5/hpf;
 1 = partial renal remission: for nephritic flare: improvement of at least 30% of serum creatinine but persistence of active urinary sediment; for proteinuric flares improvement of 50% of proteinuria;
 2 = nephritic flare: increase of 30% of serum creatinine over the basal value and active urinary sediment (>10 red blood cells/hpf, cellular casts) with or without an increase in proteinuria;
 3 = proteinuric flare: increase of proteinuria of at least 2 g/day in patients with non nephrotic syndrome or the doubling of nephrotic proteinuria with stable renal function;
 4 = persistent renal activity: the lack of achievement of remission after induction therapy.

## 3. Statistical Analysis

Mean and standard deviation, together with median and interquartile (IQ) range (25°–75° percentile) were used as descriptive statistics. For continuous variables, the nonparametric Wilcoxon test was used for assessing any difference between the two groups of patients, while the chi-square test was used for dichotomized variables. The Spearman correlation was used to analyse correlation.

Multivariate logistic regression analysis has been used to find predictors of histological classes of lupus nephritis and for the predictors of complete renal response after the beginning of induction therapy. Odds ratios (OR) and their 95% confidence interval (CI) for the covariates were derived as the antilogarithm of the regression coefficients. Multivariate linear regression analysis was used to predict the activity index at renal biopsy.

The statistical package S-Plus (MathSoft Inc.) was used for all the analyses and plots.

## 4. Results

The characteristics of the patients at renal biopsy are reported in Table 1. The 107 patients received 111 renal biopsies (4 patients, 2 biopsies). The mean age at diagnosis of SLE was 35.3 ± 14.2 years, (median 34) and that at renal biopsy was 36.4 ± 13.9 years (median 36). The mean time between the diagnosis of SLE and that of renal involvement was 5.1 ± 6.5 years, (median 3 years). In 45 patients, renal involvement was present at diagnosis of SLE.

Considering that a preliminary analysis demonstrated no significant differences in the mean values of C3, C4, anti-dsDNA, anti-C1q, anti-nucleosome, and anti-ribosome antibodies between class II and class V and between class III and Class IV LN (data not shown), the subsequent analysis was performed comparing Class II plus class V (nonproliferative forms; 26 patients) versus class III plus class IV (proliferative forms; 85 patients).

### 4.1. Prevalence of Autoantibodies and Histo Pathological Associations

At renal biopsy, high titers of anti-dsDNA were present in 77.5% of cases, high titers of anti-C1q in 70.5% of cases, high titers of anti-nucleosome antibodies in 80.3% of cases, and high titers of anti- ribosome antibodies in 14% of cases; C3 were low in 82% of cases and C4 in 74% of cases.  Table 2 reports the comparison at time of renal biopsy of clinical data and of the panel of autoantibodies between proliferative forms (class III plus class IV) and nonproliferative forms (class II plus class V) of LN. At univariate analysis, among the clinical parameters, proteinuria (*P* = 0.02) and hemoglobin (*P* = 0.0008) and among the immunological tests, C3 (*P* = 0.02) and C4 (*P* = 0.02) complement fractions, anti-DNA (*P* = 0.001), anti-C1q (*P* = 0.0005), and high titers of anti-C1q antibodies or of anti-dsDNA antibodies (*P* = 0.0000) and anti-nucleosome antibodies (*P* = 0.04) were able to differentiate proliferative from nonproliferative forms of LN. At multivariate analysis, hemoglobin (*P* = 0.008, OR = 0.68, CI: 0.52–0.9) and anti-C1q antibodies (*P* = 0.03, OR = 1.004, CI: 1.0003–1.007) were the independent predictors to discriminate between proliferative versus nonproliferative lupus nephritis. Excluding clinical parameters, at multivariate analysis, logarithm of erythrocyte sedimentation rate (ESR) (*P* = 0.03, OR = 1.9, CI: 1.08–3.42) and high titers of anti-C1q antibodies or of anti-dsDNA antibodies (*P* = 0.005, OR = 8.67, CI: 2.03–37.3) are the independent predictors which are able to discriminate proliferative from nonproliferative lupus nephritis. Among patients with proliferative forms of lupus nephritis, 95% have high titers of anti C1q or of anti-dsDNA (66.2% have high titers of both anti-C1q and of anti-dsDNA) while 5% have the results of both tests in a normal range. Among patients with nonproliferative forms, 64% have high titers of anti C1q or of anti DNA while 36% have the results of both tests in a normal range (*P* = 0.000).

The correlations among the basal clinical and the immunological data and the activity and the chronicty index at renal biopsy are reported in Table 3. All the clinical and immunological parameters evaluated with the exception for C reactive protein (CRP) and anti-ribosome antibodies showed a significant correlation with activity index. At multivariate analysis proteinuria (*P* = 0.0013), low C4 (*P* = 0.0010), and high ESR (*P* = 0,037) were the independent predictors of the activity index. Excluding the clinical variables, low C4 (*P* = 0.0004) and high ESR (*P* = 0,0025) were the independent predictors of activity index.

In contrast, serum creatinine was the only parameter among those evaluated that showed a direct correlation with the chronicity index (*R*: 0.4, *P* = 0.0000).

### 4.2. Correlations between Autoantibodies and Clinical Parameters

No correlation was found at time of renal biopsy between serum creatinine and the panel of autoantibodies, C3 and C4 complement fractions, and ESR and CRP. Among these tests, anti-C1q only showed a significant direct correlation with the amount of proteinuria (*R* = 0.2, *P* = 0.03). In addition, patients with high titers of anti-C1q or of anti-dsDNA had significant higher proteinuria (median 2.7 g/day, IQ 1.6–4.6) than those with both tests in normal range (1.8 g/day, IQ 1.0–2.2, *P* = 0.05). Anti-C1q, anti-dsDNA, and ESR were inversely correlated with hemoglobin (*R*-0.22, *P* = 0.02, *R*-0,24, *P* = 0.01, *R*-0.32, *P* = 0.002, resp.), while C3 and C4 were correlated with hemoglobin (*R* 0.36, *P* = 0.0002 and *R* 0.25, *P* = 0.01).

### 4.3. Clinical and Immunological Outcome after the Beginning of Induction Therapy

After renal biopsy and the beginning of induction therapy, 104 patients had a second evaluation between 3 and 12 months.  Table 4 reported the results of clinical and immunological tests in patients reevaluated at 3 months, at 6 months, and at 12 months. At 3 months, serum creatinine was unchanged and proteinuria did not show a significant improvement, while ESR, C3, anti-DNA, anti-C1q, and anti-nucleosome antibodies showed a significant improvement in the median values. At 6 and at 12 months, proteinuria significantly improved together with all immunological tests with the exception of ESR at 6 months and anti-ribosome antibodies at 12 months.

Altogether, during the observation period, 39 patients (37.5%) achieved and 65 (62.5%) did not achieve complete renal remission (46 were in partial remission, 13 had persistent renal activity, and 6 had persistent nephrotic syndrome). Clinical and immunological tests at the time of renal biopsy have been tested as predictors of complete renal remission (Table 5). At univariate analysis, none of the immunological tests were predictive of complete remission. At multivariate analysis, proteinuria (*P* = 0.015, OR: 0.76 CI 0.62–0.95) and the duration of therapy (*P* = 0.03 OR: 1.19 CI 1.017–1.39) were the independent predictors of complete renal remission.

## 5. Discussion

In this study, we have investigated the prevalence and the value of a panel of autoantibodies (anti-dsDNA, anti-C1q, anti-nucleosome, and anti-ribosome antibodies) as well as C3 and C4 complement fractions in predicting the activity of LN at the time of renal biopsy. The most important difference of our study compared to many previous studies is the timing of blood sampling in relation to renal activity. As a matter of fact, in the majority of the studies evaluating the predictive values of autoantibodies [11, 15, 21–23], the renal activity of LN at the time of blood sampling was judged by clinical parameters but not confirmed by renal biopsy. To the best of our knowledge, only a few studies have evaluated the association of some autoantibodies with activity of LN at the time of renal biopsy [24–27]. Trendelenburg et al. [24] reported that all but one out of 36 patients with proliferative lupus were positive for anti-C1q at the time of renal biopsy compared with 35% of patients with inactive LN. In 136 Chinese patients [25], anti-C1q and anti-dsDNA were more closely correlated with histological activity of LN at the time of renal biopsy than anti-extractable nuclear antigen antibodies, anti-C protein antibodies, anti cardiolipin, and anti Beta2 glycoprotein antibodies. The combination of anti-C1q and anti-dsDNA indicates higher renal disease activity and predicts poor long term renal outcome. Another paper [26] investigated the clinical and pathological association of anti-C1q in LN and found a higher prevalence of the autoantibody in class IV than in the other histological classes. Among the clinical variables low haemoglobin was associated with anti-C1q positivity.

In this paper, we have shown that there was a significant difference in the autoantibodies profile between proliferative forms (class III plus IV) and the other forms of LN (class V and class ll). All the autoantibodies evaluated, with the exception of anti-ribosome antibodies, had significant higher prevalence and higher titres in proliferative than in nonproliferative forms of LN. C3 and C4 complement fractions too were significantly lower in proliferative than in nonproliferative LN. At multivariate analysis, considering clinical and immunological tests, only low haemoglobin and high anti-C1q were the independent predictors of proliferative LN. Excluding the clinical variables, high ESR and positive anti-C1q or anti-dsDNA were able to discriminate between proliferative and nonproliferative LN. Ninety-five percent of patients with proliferative LN had high titers of anti C1q or of anti-dsDNA (66.2% had high titers of both tests) while 4 patients only had the results of both tests in normal range. The increasing power of the combination of anti-C1q or anti-dsDNA positivity in predicting the activity of LN has been reported by other studies [25, 28].

This higher predictive value of anti C1q for proliferative LN confirmed our findings in a previous study in which we demonstrated that 80% of flares that developed in patients with proliferative forms were associated with high titres of anti-C1q in comparison to only 54% of those that occurred in the nonproliferative forms [16]. Instead, other cohort studies did not show differences in the prevalence of antiC1q between proliferative and nonproliferative lupus nephritis [28–30]. This discrepancy could be due to the fact that in these studies the diagnosis of renal activity was done on clinical grounds and not confirmed by renal biopsy. Again, anti-C1q, alone or associated with anti-dsDNA, was the only test among the immunological parameters that significantly correlated with the amount of proteinuria. None of the immunological tests correlated with serum creatinine but the majority of our patients had normal renal function, and many tests correlated with hemoglobin, a manifestation not specific for LN but an expression of the general activity of SLE. Low C4 and high ESR were the independent predictors of a high activity index at multivariate analysis while none of the tests of the panel correlated with chronicity index.

In contrast to what was reported by Yang et al. [25], in our cohort, none of the immunological tests at the time of renal biopsy was predictive of the renal response, at least in the short term. However, we have shown that, three months after the start of the induction therapy and prior to the improvement of proteinuria, a significant reduction of the mean value of anti-C1q, anti-dsDNA, and anti-nucleosome antibody occurred. The progressive and significant drop in autoantibodies titres continued at 6 and at 12 months together with a clinical improvement as reported in other studies [24, 26, 31].

Anti-C1q antibodies can be detected by different methods (reviewed in [32, 33]). In the early 1980s, a solid-phase assay using purified C1q, immobilized on plastic assay plates, was used for the detection of circulating immune complexes in SLE patients. To differentiate between immune complexes and anti-C1q antibodies, high-salt concentrations (0.5–1.0 M sodium chloride) were used. Using this method, the binding of the globular heads of C1q to immune complexes is prevented, whereas anti-C1q antibodies can still interact with the coated C1q. Subsequently, to eliminate the need to use high-ionic strength buffer, assays have been developed that utilize only the C1q collagen-like region. The use of the purified collagen-like region may potentially be more reliable. However, additional exposed epitopes, by cleaving of the C1q molecule, might interfere with the results obtained with this assay. More recently, peptides derived from C1q that have the properties to detect a major linear epitope in a high percentage of the patients in the absence of high-ionic strength buffer has been proposed [33]. Unfortunately, systematic studies comparing anti-C1q antibody detected by different assays are not available and different studies have used different methods. In our study, we have used the “classic” assay which has been used in the majority of published clinical studies because it is more readily available.

## 6. Conclusions

We found a significantly different autoantibodies profile between proliferative and nonproliferative forms of LN at the time of renal biopsy. Among the panel of autoantibodies evaluated in this study, anti-C1q alone or in combination with anti-dsDNA emerged as the most reliable in differentiating proliferative and nonproliferative LN and anti-C1q is the only test correlated with the clinical presentation of LN. After the beginning of therapy, the titer of the autoantibodies progressively and significantly reduced, but none of them was predictive of complete renal remission. The results of this work, which outlines the role of autoantibodies and in particular of anti-C1q, in defining the activity of lupus nephritis at the time of renal biopsy, confirm their utility in diagnosing the acute exacerbations of LN made on clinical grounds only.

## Figures and Tables

**Table 1:** Clinical and histological characteristics at renal biopsy of the 107 lupus nephritis patients enrolled in the study who received 111 renal biopsies.

Males/females	13/94
Age at diagnosis of renal biopsy (M ± SD)	36.4 ± 13.9
Duration of SLE years (M ± SD)	5.1 ± 6.5
Serum creatinine mg/dL (M ± SD)	1.07 ± 0.76
Number of patients with serum creatinine >1.2 mg/dL	31 (28%)
Proteinuria g/24 h (M ± SD)	3.4 ± 2.85
Number of patients with nephrotic syndrome	44 (39.6%)
Hemoglobin g/dL	11.53 ± 1.9
Class II (number of patients)/activity index/chronicity index	8 (7.2%)/1.6 ± 3.2/0.4 ± 0.9
Class III (number of patients)/activity index/chronicity index^*^	35 (31.5%)/5.3 ± 2.5/1.7 ± 1.74
Class IV (number of patients)/activity index/chronicity index^**^	50 (45%)/9.0 ± 3.2/2.1 ± 1.7
Class V (number of patients)/activity index/chronicity index	18 (16.2%)/1.1 ± 2.1/0.7 ± 1.2
Methylprednisolone pulses 0.5–1 g/day for 3 days^***^	80 (72%)
Oral prednisone 1 mg/kg/day for 1 month	31 (28%)
Oral cyclophosphamide 1-2 mg/kg/day for 2-3 months^§^	36 (32%)
6 fortnightly cyclophosphamide pulses of of 0.5 g^§§^	11 (10%)
6 monthly cyclophosphamide pulses of 1 g/m^2^^§§§^	18 (16%)
Mycophenolate mofetil 2 g/day^$^	20 (18%)
Maintenance therapy: mycophenolate mofetil/azathioprine	30/21

M, mean; SD, standard deviation. ^*^including 15 patients with class III + V; ^**^including 15 patients with class IV + V; ^***^(1 patient of class II, 27 of class III, 45 of class IV, and 7 of class V); ^§^(14 patients of class III and 22 of class IV); ^§§^(3 patients of class III, 6 of class IV, and 2 of class V); ^§§§^(2 patients of class II, 5 of class III, 10 of class IV, and 1 of class V), ^$^(2 patients of class II, 5 of class III, 8 of class IV, and 5 of class V).

**Table 2:** Comparison of clinical and immunological features between nonproliferative forms (class II + class V) and proliferative forms (class III + class IV) lupus nephritis.

	Non proliferative LN 26 patients	Proliferative LN 85 pateints	*P*
Serum creatinine mg/dL	0.8 (0.67–0.96)	0.9 (0.74–1.15)	Ns
Proteinuria g/day	1.94 (1–2.74)	2.8 (1.6–4.7)	0.02
Hemoglobin g/dL	12.9 (11.3–14)	11.2 (9.8–12.3)	0.0008
Activity index	1 (0–3)	7 (5–9)	0.0000
Chronicity index	1 (0–2)	2 (1–3)	0.02
Erythrocyte sedimentation rate mm	21 (11–46.5)	37.5 (20.7–71.5)	0.07
C reactive protein mg/dL	0.39 (0.08–0.89)	0.3 (0.11–0.6)	Ns
C3 mg/dL	72 (60–94)	62 (46.7–80.5)	0.02
C3 <90 mg/dL	65%	87.3%	0.08
C4 mg/dL	13 (9–19)	8.5 (5–14)	0.02
C4 <15 mg/dL	57.6%	78.8%	0.01
Anti-DNA antibodies U/mL	67 (18–135)	183 (85–400)	0.001
Anti-DNA antibodies U/mL >50	50%	86%	0.001
Anti-C1q antibodies AU	41 (22–123)	216 (63–320)	0.0005
Anti-C1q antibodies AU >55	44%	79%	0.003
Anti-nucleosome Ab U	51 (18.5–97)	95 (44–118)	0.04
Anti-nucleosome antibodies Ab U >20	68%	84%	0.02
Anti-DNA >50 U/mL or anti-C1q >55 AU	9 (36%)	80 (95%)	0.000
Anti-ribosome antibodies	3 (2–5.5)	2 (2–6)	Ns
Anti-ribosome antibodies >20	16%	13%	Ns
Lupus anti-coagulant positivity	22.7%	15,4%	Ns
Antiphospholipid antibodies	32%	25%	Ns

The data are reported as Median and interquartile ranges.

**Table 3:** Correlations of activity and chronicity index with clinical and immunological tests.

	Activity index *R*	*P*	Chronicity index *R*	*P*
Serum creatinine mg/dL	0.23	0.017	0.4	0.0000
Proteinuria g/day	0.3	0.0014	0.17	0.08
Erythrocyte sedimentation rate mm	0.24	0.02	Ns	Ns
C reactive protein mg/dL	Ns	Ns	Ns	Ns
C3 mg/dL	−0.34	0.0003	Ns	Ns
C4 mg/dL	−0.28	0.004	Ns	Ns
Anti-DNA antibodies U/mL	0.31	0.001	Ns	Ns
Anti-C1q antibodies AU	0.24	0.01	Ns	Ns
Anti-nucleosome antibodies Ab U	0.29	0.041	Ns	Ns
Anti-Ribosome antibodies	Ns	Ns	Ns	Ns
Lupus anticoagulant	Ns	Ns	Ns	Ns
Antiphospholipid antibodies	Ns	Ns	Ns	Ns

**Table 4:** Outcome of clinical and immunological parameters at 3, 6, and 12 months after the beginning of induction therapy.

	Basal 16 pts	At 3 months	*P*	Basal 46 pts	At 6 months	*P*	Basal 42 pts	At 12 months	*P*
S. Creat. mg/dL	0.76 0.68–1.6	0.76 0.65–0.89	Ns	0.87 0.7–1	0.85 0.7–1	Ns	0.82 0.67–0.96	0.74 0.67–0.93	Ns

Proteinuria g/day	2.5 1.8–4	0.94 0.52–4.2	Ns	2.2 1.3–3.9	0.92 0.3–1.5	0.0000	3.0 2.05–4.9	0.51 0.23–1.26	0.0000

ESR mm	65 25.7–82	36.5 10.5–51.7	0.04	29.5 12–63.7	21.5 14.2–31.2	Ns	33 20–67	13.5 10.2–32.7	0.0003

CRP mg/dL	0.47 0.261.1	0.30 0.2–1.32	Ns	0.29 0.09–0.7	0.1 0.06–0.28	0.005	0.31 0.12–0.61	0.1 0.04–0.2	0.005

C3 mg/dL	67 60–87	83 74.5–100.5	0.03	73 47.2–87.7	92 79–108	0.0000	63 48–81.2	86.5 71–101.5	0.0000

C4 mg/dL	10 8–13	13 9–20	Ns	10 5.2–16.7	17 11–21	0.0000	9 5–14.7	13.5 10–20	0.001

Anti-DNA Ab UmL	79.5 10.0–291	38 11.7–85	0.01	173 69.5.400	71.7 15.7–84.7	0.0000	124 83.7–345	48 34–104	0.0000

Anti-C1q Ab AU	120.5 52–315	52.5 39–121	0.002	94 35–234	50 22.7–108.5	0.0003	147 56–320	65 35.7–122	0.0000

Anti-nucleosome Ab	66 42.7–110	58 39–83	0.04	97.5 30–118	41 13–56	0.0001	88 51–109	32 16–70	0.0001

Anti-ribosome Ab	2.5 1.75–4	2 2-3	Ns	3.5 2–6	2 2–3.5	0.0007	2 2–10	2 2-3	Ns

The data are reported as Median and interquartile ranges: S. = serum, Creat. = creatinine, ESR = erythrocyte sedimentation rate, CPR = C reactive protein, and Ab = antibodies.

**Table 5:** Predictors of complete renal remissions among the clinical and the immunological tests at renal biopsy.

	Complete remission 39 patients	No complete remission 65 patients	*P*
Months of therapy	6.0 (5.2–7.4)	5.2 (3.5–6.8)	0.02
Serum creatinine mg/dL	0.83 (0.64–0.95)	0.85 (0.7–1.26)	0.15
Proteinuria g/day	1.96 (12–2.76)	3.45 (2.1–5)	0.0009
Hemoglobin g/dL	12.0 (11.0–12.9)	11.6 (10–12.7)	0.12
Erythrocyte sedimentation rate	33 (17.5–61.2)	39 (16–73)	0.7
C reactive protein mg/dL	0.3 (0.09–1.22)	0.3 (0.14–0.67)	0.6
C3 mg/dL	66 (48–82.2)	70 (48–88)	0.5
C4 mg/dL	9 (5.2–115.7)	10 (5–14)	0.7
Anti DNA antibodies U/mL	147 (83.5–380)	132 (43–400)	0.5
Anti C1q antibodies AU	120 (35–320)	128 (42.5–320)	0.5
Anti-nucleosome antibodies U	88 (57–111)	92.5 (33.2–115.7)	0.8
Anti-DNA >55 U/mL or anti-C1q >50 AU antibodies	34 (87%)	58 (89,2%)	1
Anti-ribosome antibodies	2 (2–5.5)	2 (2–5.7)	0.1

The data are reported as Median and interquartile ranges.
